# Increased Surgical Complications but Improved Overall Survival with Adult Living Donor Compared to Deceased Donor Liver Transplantation: A Systematic Review and Meta-Analysis

**DOI:** 10.1155/2020/1320830

**Published:** 2020-08-24

**Authors:** Wei Tang, Jian-Guo Qiu, Yang Cai, Luo Cheng, Cheng-You Du

**Affiliations:** Department of Hepatobiliary Surgery, The First Affiliated Hospital of Chongqing Medical University, Chongqing 400016, China

## Abstract

**Background:**

Living donor liver transplantation (LDLT) provides an alternative to deceased donor liver transplantation (DDLT) for patients with end-stage liver disease in the circumstance of scarcity of deceased grafts. However, the outcomes of LDLT remain controversial.

**Method:**

A systematic review and meta-analysis were performed to compare the outcomes of LDLT with DDLT. Twelve outcomes were assessed.

**Results:**

Thirty-nine studies involving 38563 patients were included. LDLT was comparable in red blood cell transfusion, perioperative mortality, length of hospital stay, retransplantation rate, hepatitis C virus recurrence rate, and hepatocellular carcinoma recurrence rate with DDLT. Cold ischemia time was shorter and duration of recipient operation was longer in LDLT. Postoperative intra-abdominal bleeding rate occurred less frequently in LDLT recipients (odds ratio (OR) = 0.64, 95%confidence interval (CI) = 0.46 − 0.88, *P* = 0.006), but this did not decrease the perioperative mortality. LDLT was associated with significantly higher biliary (OR = 2.23, 95%CI = 1.59 − 3.13, *P* < 0.00001) and vascular (OR = 2.00, 95%CI = 1.31 − 3.07, *P* = 0.001) complication rates and better overall survival (OS) (1 year: OR = 1.32, 95%CI = 1.01 − 1.72, *P* = 0.04; 3 years: OR = 1.39, 95%CI = 1.14 − 1.69, *P* = 0.0010; and 5 years: OR = 1.33, 95%CI = 1.04 − 1.70, *P* = 0.02). According to subgroup analysis, biliary complication rate and OS improved dramatically as experience increased, while vascular complication rate could not be improved because it was mainly caused by the difference of the donor type itself.

**Conclusions:**

LDLT remains a valuable option for patients in need of liver transplantation for it provides an excellent alternative to DDLT without compromising recipient outcomes. Further refinement in biliary and vascular reconstruction techniques and the accumulation of liver transplantation centers' experience are the key factors in expanding the application of LDLT.

## 1. Introduction

Liver transplantation (LT) is a well-established therapeutic option for patients with irremediable end-stage liver disease. However, the worldwide scarcity of deceased donor livers is the greatest challenge nowadays. Nearly a quarter of patients with liver failure died while waiting for liver grafts [1–4]. Fortunately, in 1966, the idea of living donor liver transplantation (LDLT) was aroused [5], and in 1989, the first successful LDLT was performed [6]. After decades of development, LDLT has been widely considered as an alternative to deceased donor liver transplantation (DDLT). Compared with DDLT, it is generally accepted by us that LDLT has shorter waiting time, younger donor age, and better organization of surgery time. Oppositely, LDLT is criticized for its smaller graft volume, donor risk, and ethical controversies. The efficacy of LDLT versus DDLT is controversial. Although lots of studies comparing the clinical outcomes of LDLT with DDLT were carried out in the past decades, the issues remained for the inconformity of conclusions. Whether a distinct disparity exists between these 2 therapeutic regimens and which type of LT can obtain better clinical outcomes need further investigation. Therefore, with the aim of comparing the outcomes of LDLT with DDLT, we systematically summarized the current available data and performed a meta-analysis.

## 2. Materials and Methods

### 2.1. Literature Search and Study Selection

We adhered to the 2009 preferred reporting items for systematic reviews and meta-analysis statement. To provide an adequate overview of the current literature, databases of PubMed, Embase, and the Cochrane Library from inception to 21 November 2019 were chosen for screening; an additional search with Google Scholar was performed to supplement the primary search. A combination of the following terms was used as a strategy of literature search: living donor, deceased donor, cadaveric donor, CLT (cadaveric liver transplantation), LDLT, DDLT, and liver transplantation. Two authors (Tang and Qiu) carried out the search independently and any discrepancies regarding the study selection were resolved by them. No restriction of language or publication type was set in the search. The study protocol was approved by the Science and Research Office of the First Affiliated Hospital of Chongqing Medical University.

### 2.2. Inclusion and Exclusion Criteria

The inclusion criteria were (1) published prospective or retrospective cohort studies and randomized controlled trials, (2) studies comparing LDLT with DDLT in adult patients, and (3) studies with at least 1 of the aforementioned outcomes. The exclusion criteria were (1) case reports, reviews, letters, editorials, and conference reports; (2) studies lacking a control group; (3) studies without available data; (4) studies investigating emergency LT; (5) studies without a clear description of methods or baseline characteristics; and (6) studies with less than 100 recipients in total. Moreover, for studies from the same database, only the one with the largest sample size was included.

### 2.3. Outcomes of Interest

We assessed 12 outcomes of LT in this meta-analysis, including cold ischemia time (CIT), amount of allogeneic red blood cell (RBC) transfusion, duration of the recipient operation (DRO), postoperative intra-abdominal bleeding rate, perioperative mortality, length of hospital stay, vascular complication rate, biliary complication rate, retransplantation rate, hepatitis C virus (HCV) recurrence rate, hepatocellular carcinoma (HCC) recurrence rate, and patient overall survival (OS).

### 2.4. Quality Assessment and Data Extraction

The methodological quality of each included study was assessed using the Newcastle-Ottawa quality assessment scale [7] by 2 reviewers (Cai and Cheng) independently. The extracted data included general information (first author, year of publication, source journal, country, study design, sample size, diagnoses of patients, recipient and donor age, follow-up period, gender, and the source of clinical data) and 12 outcomes (CIT, RBC transfusion, DRO, postoperative intra-abdominal bleeding rate, perioperative mortality, length of hospital stay, vascular complication rate, biliary complication rate, retransplantation rate, HCV recurrence rate, HCC recurrence rate, and OS). The obtained data were then compared by the reviewers, inconsistencies were discussed, and a third reviewer (Du) was consulted to reach a consensus if necessary.

### 2.5. Statistical Analysis

The meta-analysis was carried out in accordance with the Cochrane Reviewer's Handbook, and statistical analyses were performed with the Review Manager (RevMan) software (Version 5.3, The Cochrane Collaboration, The Nordic Cochrane Center, Copenhagen, Denmark). The results were presented by odds ratio (OR) with 95% confidence interval (CI) for dichotomous data and mean difference (MD) with 95% CI for continuous data. For data which were reported as medians and ranges, the methods described by Hozo et al. [8] and Higgins and Green [9] were used to transform into mean and standard deviation. Heterogeneity among studies was estimated using the Chi^2^-square test (*P* < 0.10 represented statistically significant heterogeneity) and the *I*^2^ test (*I*^2^ > 50% represented statistically significant heterogeneity). When indicating no significant heterogeneity, a fixed-effect model was used. Otherwise, a random-effect model was used and a subgroup analysis was performed to explore the discrepancy. Funnel plots were performed to assess the publication bias, and the bias was excluded if a symmetrical distribution was shown. Moreover, a sensitivity analysis was performed by removing each study in turn to evaluate the stability of a pooled estimate. Pooled analyses were visualized with forest plots, and statistical significance was considered at *P* < 0.05.

## 3. Results

### 3.1. Study Characteristics and Quality Assessment

**Figure 1:**
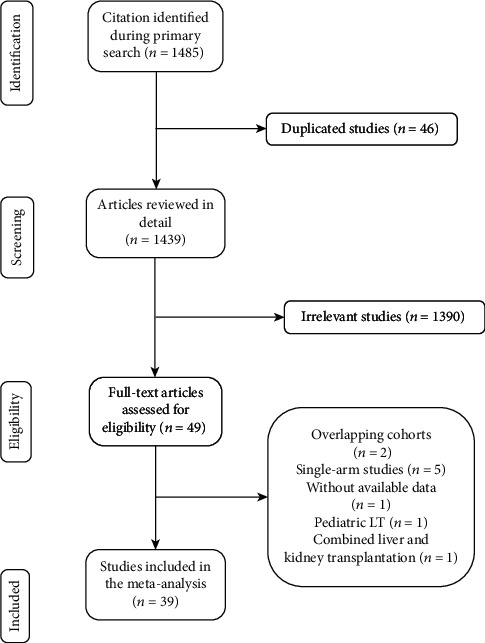
Flow diagram showing process of literature search and study selection.

As shown in a flow diagram (Figure 1), 1439 articles were found using a combination of search terms and 1390 irrelevant articles were excluded according to the inclusion and exclusion criteria after screening titles and abstracts. Then, 10 studies were excluded after full text review for the following reasons: 2 studies were based on overlapping data from the same database, 5 studies lacked a control group, 1 study had no available data, 1 study focused on pediatric LT, and 1 study was about combined liver and kidney transplantation. Finally, 39 studies [4, 10–47] (7 prospective cohorts and 32 retrospective cohorts) were included in the meta-analysis.

The characteristics of the 39 included studies are presented in Table 1. All studies were well designed to compare two arms: LDLT and DDLT. The follow-up period ranged from 1988 to 2019. Fifteen studies investigated patients with various liver diseases, 10 studies were about hepatitis B/C virus-related diseases, and 14 studies focused on HCC. Three studies [21, 37, 40] were derived from the Adult-to-Adult Living Donor Liver Transplantation Cohort Study, 3 studies [20, 39, 46] were based on the United Network for Organ Sharing, 1 study [18] was derived from the University Health System Consortium and Scientific Registry of Transplant Recipients, 1 study [14] was based on the China Liver Transplant Registry, and the rest of the studies were derived from a single center or from multiple institutions. These studies were conducted in the east (*n* = 14) and in the west (*n* = 25). Most of the included studies showed satisfactory quality with selection criteria, comparability of patient characteristics, and adequate follow-up. All cohorts got 6 or more stars (Table 2).

### 3.2. CIT

**Figure 2:**
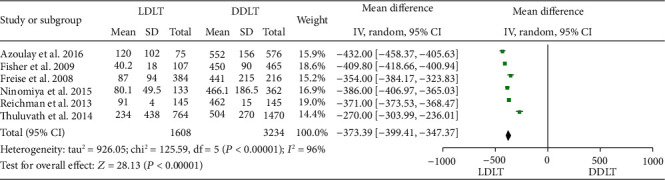
Meta-analysis of studies comparing CIT between LDLT and DDLT recipients based on a random-effect model. CIT: cold ischemia time; LDLT: living donor liver transplantation; DDLT: deceased donor liver transplantation.

Six studies [11, 16, 20, 31, 32, 40] reported CIT, and all six of them suggested it was shorter in LDLT. There was significant heterogeneity (*P* < 0.00001, *I*^2^ = 96%). A random-effect model indicated a significant difference between LDLT and DDLT, and CIT of LDLT was much shorter than DDLT (WMD = −373.39, 95%CI = −399.41 to -347.37, *P* < 0.00001; Figure 2).

### 3.3. RBC Transfusion

**Figure 3:**
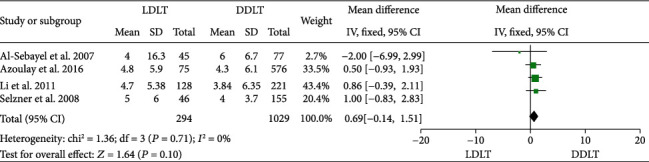
Meta-analysis of studies comparing RBC transfusion between LDLT and DDLT recipients based on a fixed-effect model. RBC: red blood cell; LDLT: living donor liver transplantation; DDLT: deceased donor liver transplantation.

Four studies [32, 38, 43, 45] reported RBC transfusion, with all four of them indicating no significant difference between LDLT and DDLT. No significant heterogeneity was observed (*P* = 0.71, *I*^2^ = 0%). A fixed-effect model was used, and pooled results showed that LDLT was comparable with DDLT in RBC transfusion (WMD = 0.69, 95%CI = −0.14 to 1.51, *P* = 0.10; Figure 3).

### 3.4. DRO

**Figure 4:**
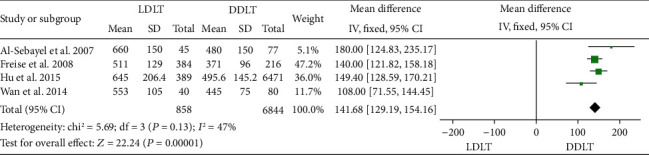
Meta-analysis of studies comparing DRO between LDLT and DDLT recipients based on a fixed-effect model. DRO: duration of the recipient operation; LDLT: living donor liver transplantation; DDLT: deceased donor liver transplantation.

DRO was reported in four studies [14, 34, 40, 45], and all four of them showed that it was significantly longer in LDLT. No significant heterogeneity was observed (*P* = 0.13, *I*^2^ = 47%). A fixed-model was used, and the pooled DRO of LDLT was found to be significantly longer than that of DDLT (WMD = 141.68, 95%CI = 129.19 to 154.16, *P* < 0.00001; Figure 4).

### 3.5. Postoperative Intra-Abdominal Bleeding Rate

**Figure 5:**
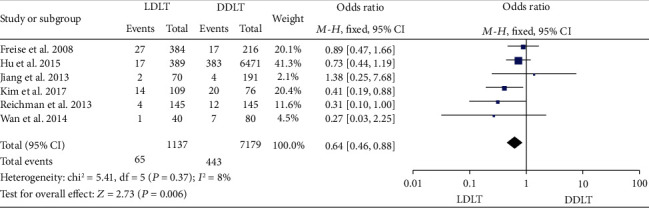
Meta-analysis of studies comparing postoperative intra-abdominal bleeding rate between LDLT and DDLT recipients based on a fixed-effect model. LDLT: living donor liver transplantation; DDLT: deceased donor liver transplantation.

Six studies [12–14, 31, 34, 40] reported the postoperative intra-abdominal bleeding rate. While five of them [12, 14, 31, 34, 40] indicated no significant difference between LDLT and DDLT, 1 study [14] showed a significantly lower intra-abdominal bleeding rate in LDLT. Notably, pooled results with a fixed-effect model revealed that the intra-abdominal bleeding rate of LDLT was significantly lower than that of DDLT (OR = 0.64, 95%CI = 0.46 to 0.88, *P* = 0.006; Figure 5). Moreover, no significant heterogeneity was observed (*P* = 0.37, *I*^2^ = 8%).

### 3.6. Perioperative Mortality

**Figure 6:**
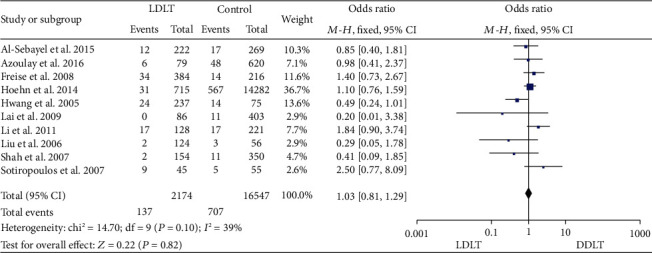
Meta-analysis of studies comparing perioperative mortality between LDLT and DDLT recipients based on a fixed-effect model. LDLT: living donor liver transplantation; DDLT: deceased donor liver transplantation.

Ten studies [18, 25–27, 32, 36, 38, 40–42] reported perioperative mortality, and all of them suggested no significant difference between LDLT and DDLT. No significant heterogeneity was observed (*P* = 0.10, *I*^2^ = 39%). A fixed-effect model suggested comparable perioperative mortality between LDLT and DDLT (OR = 1.03, 95%CI = 0.81 to 1.29, *P* = 0.82; Figure 6).

### 3.7. Length of Hospital Stay

**Figure 7:**
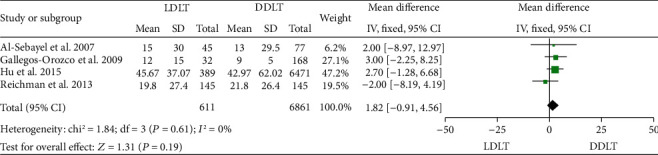
Meta-analysis of studies comparing length of hospital stay between LDLT and DDLT recipients based on a fixed-effect model. LDLT: living donor liver transplantation; DDLT: deceased donor liver transplantation.

Four studies [14, 31, 35, 45] reported the length of hospital stay, and all of them showed that no significant difference existed between LDLT and DDLT. A fixed-effect model revealed a similar length of hospital stay between LDLT and DDLT (WMD = 1.82, 95%CI = −0.91 to 4.56, *P* = 0.19; Figure 7). There was no significant heterogeneity observed (*P* = 0.61, *I*^2^ = 0%).

### 3.8. Vascular Complication Rate

**Figure 8:**
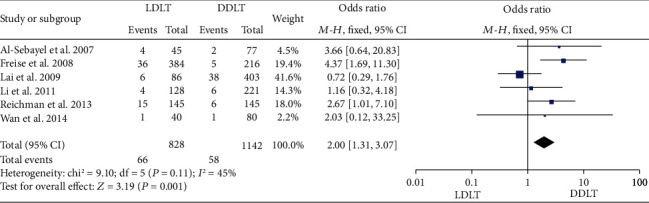
Meta-analysis of studies comparing the vascular complication rate between LDLT and DDLT recipients based on a fixed-effect model. LDLT: living donor liver transplantation; DDLT: deceased donor liver transplantation.

Six studies [31, 34, 38, 40, 42, 45] reported the vascular complication rate. While five of them [31, 34, 38, 42, 45] suggested no significant difference between LDLT and DDLT, 1 study [40] showed a significantly higher vascular complication rate in LDLT. There was no significant heterogeneity (*P* = 0.11, *I*^2^ = 45%). A fixed-effect model showed a significantly higher vascular complication rate in LDLT (OR = 2.00, 95%CI = 1.31 to 3.07, *P* = 0.001; Figure 8).

### 3.9. Biliary Complication Rate

**Figure 9:**
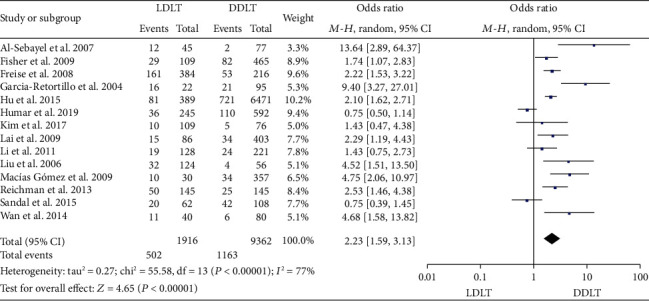
Meta-analysis of studies comparing the biliary complication rate between LDLT and DDLT recipients based on a random-effect model. LDLT: living donor liver transplantation; DDLT: deceased donor liver transplantation.

Fourteen studies [10, 11, 13, 14, 17, 19, 23, 31, 34, 36, 38, 40, 42, 45] reported the biliary complication rate. Four studies [10, 13, 17, 38] showed no significant difference between LDLT and DDLT, while the rest of the studies [11, 14, 19, 23, 31, 34, 36, 40, 42, 45] indicated a significantly higher biliary complication rate in LDLT. Significant heterogeneity existed (*P* < 0.00001, *I*^2^ = 77%). A random-effect model revealed a significantly higher biliary complication rate in LDLT (OR = 2.23, 95%CI = 1.59 to 3.13, *P* < 0.00001; Figure 9).

### 3.10. Retransplantation Rate

**Figure 10:**
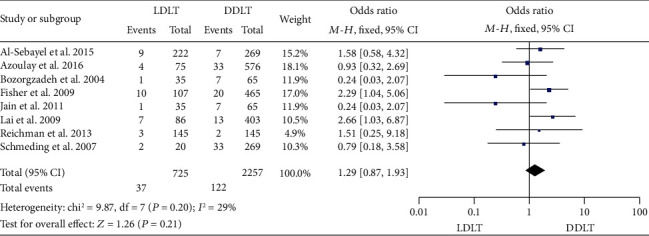
Meta-analysis of studies comparing the retransplantation rate between LDLT and DDLT recipients based on a fixed-effect model. LDLT: living donor liver transplantation; DDLT: deceased donor liver transplantation.

Eight studies [11, 15, 22, 24, 27, 31, 32, 42] reported the retransplantation rate. While six of them [15, 22, 24, 27, 31, 32] indicated no significant difference between LDLT and DDLT, 2 studies [11, 42] showed a significantly higher retransplantation rate in LDLT. No significant heterogeneity was observed (*P* = 0.20, *I*^2^ = 29%). A fixed-effect model showed a comparable retransplantation rate between LDLT and DDLT (OR = 1.29, 95%CI = 0.87 to 1.93, *P* = 0.21; Figure 10).

### 3.11. HCV Recurrence Rate

**Figure 11:**
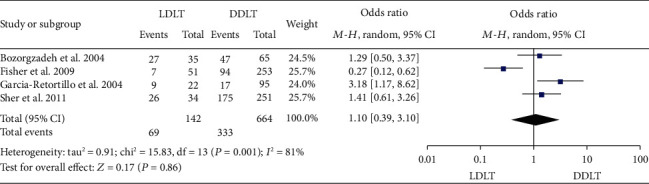
Meta-analysis of studies comparing HCV recurrence rate between LDLT and DDLT recipients based on a random-effect model. HCV: hepatitis C virus; LDLT: living donor liver transplantation; DDLT: deceased donor liver transplantation.

Four studies [11, 23, 24, 46] reported the HCV recurrence rate. Two of them [24, 46] suggested no significant difference between LDLT and DDLT, 1 [23] showed a significantly higher HCV recurrence rate in LDLT, and 1 [11] showed a lower HCV recurrence rate in LDLT. Significant heterogeneity was observed (*P* = 0.001, *I*^2^ = 81%). A random-effect model suggested no significant difference between LDLT and DDLT (OR = 1.10, 95%CI = 0.39 to 3.10, *P* = 0.86; Figure 11).

### 3.12. HCC Recurrence Rate

**Figure 12:**
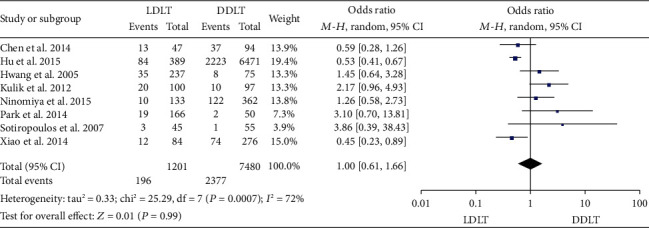
Meta-analysis of studies comparing 1-year HCC recurrence rate between LDLT and DDLT recipients based on a random-effect model. HCC: hepatocellular carcinoma; LDLT: living donor liver transplantation; DDLT: deceased donor liver transplantation.

**Figure 13:**
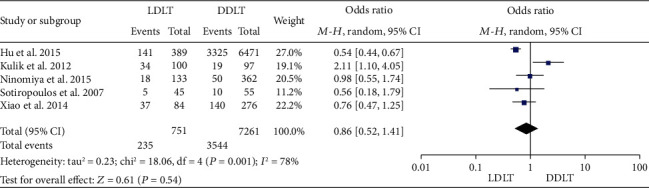
Meta-analysis of studies comparing 3-year HCC recurrence rate between LDLT and DDLT recipients based on a random-effect model. HCC: hepatocellular carcinoma; LDLT: living donor liver transplantation; DDLT: deceased donor liver transplantation.

**Figure 14:**
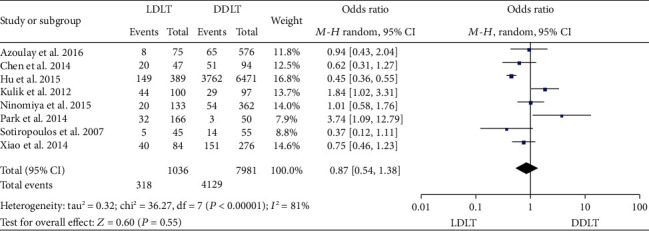
Meta-analysis of studies comparing 5-year HCC recurrence rate between LDLT and DDLT recipients based on a random-effect model. HCC: hepatocellular carcinoma; LDLT: living donor liver transplantation; DDLT: deceased donor liver transplantation.

Eight studies [14, 16, 25, 26, 28, 33, 37, 44] reported the 1-year HCC recurrence rate; while six of them [16, 25, 26, 33, 37, 44] showed no significant difference between LDLT and DDLT, 2 [14, 28] suggested a significantly lower recurrence rate in LDLT. Five studies [14, 16, 26, 28, 37] reported the 3-year HCC recurrence rate; three of them [16, 26, 28] indicated no significant difference between LDLT and DDLT, 1 [14] showed a significantly lower recurrence rate in LDLT, and 1 [37] suggested a higher recurrence rate in LDLT. Eight studies [14, 16, 26, 28, 32, 33, 37, 44] reported the 5-year HCC recurrence rate; 5 of them [16, 26, 28, 32, 44] indicated no significant difference between LDLT and DDLT, 2 [33, 37] showed a significantly higher recurrence rate in LDLT, and 1 [14] suggested a lower recurrence rate in LDLT. Significant heterogeneity was observed (1 year: *P* = 0.0007, *I*^2^ = 72%; 3 years: *P* = 0.001, *I*^2^ = 78%; and 5 years: *P* < 0.00001, *I*^2^ = 81%). Random-effect models indicated comparable 1-, 3-, and 5-year HCC recurrence rates between LDLT and DDLT (1 year: OR = 1.00, 95%CI = 0.61 to 1.66, *P* = 0.99, see Figure 12; 3 years: OR = 0.86, 95%CI = 0.52 to 1.41, *P* = 0.54, see Figure 13; and 5 years: OR = 0.87, 95%CI = 0.54 to 1.38, *P* = 0.55, see Figure 14).

### 3.13. OS

**Figure 15:**
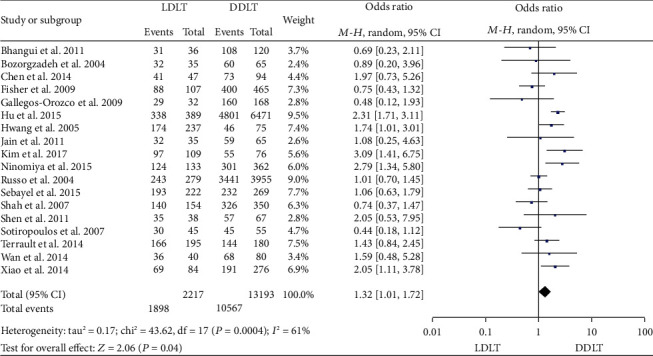
Meta-analysis of studies comparing 1-year OS between LDLT and DDLT recipients based on a random-effect model. OS: overall survival; LDLT: living donor liver transplantation; DDLT: deceased donor liver transplantation.

**Figure 16:**
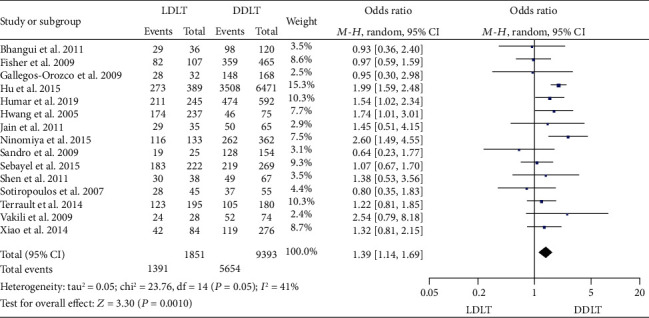
Meta-analysis of studies comparing 3-year OS between LDLT and DDLT recipients based on a random-effect model. OS: overall survival; LDLT: living donor liver transplantation; DDLT: deceased donor liver transplantation.

**Figure 17:**
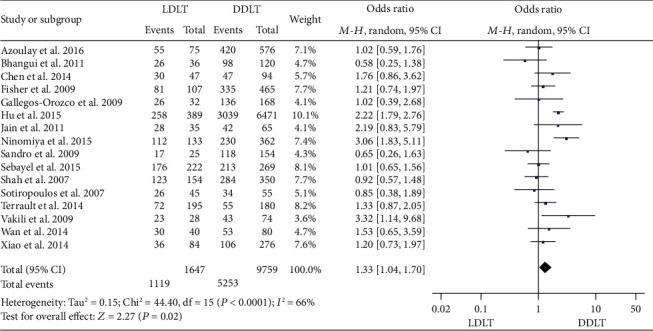
Meta-analysis of studies comparing 5-year OS between LDLT and DDLT recipients based on a random-effect model. OS: overall survival; LDLT: living donor liver transplantation; DDLT: deceased donor liver transplantation.

Eighteen studies [4, 11, 13–16, 21, 24–28, 34, 35, 39, 41, 44, 47] reported 1-year OS. Four of them [13, 14, 16, 28] suggested a significantly higher 1-year OS in LDLT, and the rest [4, 11, 15, 21, 24–27, 34, 35, 39, 41, 44, 47] showed no significant difference between LDLT and DDLT. A random-effect model revealed a significantly higher 1-year OS in LDLT (OR = 1.32, 95%CI = 1.01 to 1.72, *P* = 0.04; Figure 15). Fifteen studies [4, 10, 11, 14–16, 21, 25–30, 35, 47] reported 3-year OS. Two [14, 16] suggested a significantly higher 3-year OS in LDLT, and the rest [4, 10, 11, 15, 21, 25–30, 35, 47] showed no significant difference between LDLT and DDLT. A random-effect model revealed a significantly higher 3-year OS in LDLT (OR = 1.39, 95%CI = 1.14 to 1.69, *P* = 0.0010; Figure 16). Sixteen studies [4, 11, 14–16, 21, 26–30, 32, 34, 35, 41, 44] reported 5-year OS. Three studies [14, 16, 29] suggested a significantly higher 5-year OS in LDLT, and the rest [4, 11, 15, 21, 26–28, 30, 32, 34, 35, 41, 44] showed comparable OS between LDLT and DDLT. A random-effect model revealed a significantly higher 5-year OS in LDLT (OR = 1.33, 95%CI = 1.04 to 1.70, *P* = 0.02; Figure 17). Significant heterogeneity was observed (1 year: *P* = 0.0004, *I*^2^ = 61%; 3 years: *P* = 0.05, *I*^2^ = 41%; and 5 years: *P* < 0.0001, *I*^2^ = 66%).

### 3.14. Subgroup Analysis

To investigate the source of heterogeneity among studies, a subgroup analysis was carried out by stratifying the analysis according to several important factors, including study design, sample size, transplantation area, and patient diagnosis (Table 3). Moreover, to probe into the effect of sample size on the vascular complication rate, a subgroup was performed for it through insignificant heterogeneity. Most subgroup results were in line with the main results, while stratification of OS showed several points of discordance. Notably, according to the subgroup analysis, the ORs of OS were significantly higher and the OR of the biliary complication rate was significantly lower in subgroups with a bigger sample size, while this phenomenon could not be observed in the vascular complication rate. It indicated that OS and the biliary complication rate might dramatically improve as the centers' experience increased, while the disparity between the vascular complication rate of LDLT and that of DDLT was mainly caused by the difference of the donor type itself, and thus, it could not be improved with an accumulation of experience.

### 3.15. Publication Bias Assessment and Sensitivity Analysis

There was no evidence of publication bias for RBC transfusion, DRO, postoperative intra-abdominal bleeding rate, perioperative mortality, length of hospital stay, vascular complication rate, retransplantation rate, HCV recurrence rate, HCC recurrence rate (5-year), and OS, with a symmetrical appearance on funnel plots. For CIT, biliary complication rate and HCC recurrence rate (1- and 3-year), funnel plots showed an asymmetry which suggested that negative studies might be less reported. According to the sensitivity analysis, most of the overall results did not change after the exclusion of a single study except vascular complication rate, HCC recurrence (3-year), and OS (1- and 5-year).

## 4. Discussion

LDLT, which can provide a large pool of organs, is widely perceived as an alternative to DDLT for overcoming the scarcity of liver grafts. The survival of patients with end-stage liver diseases has been hugely improved with the advent of LDLT in the past decades. Furthermore, for emergency patients with fulminant hepatic failure, LDLT is also the optimal choice to timely save the patients' lives given the lack of deceased donor grafts [48]. However, LDLT involves a healthy donor, and the median mortality and morbidity of a donation are 0.2% and 16.1% [49]. It brings up controversies and ethical problems. Whether LDLT can provide comparable or better outcomes than DDLT is particularly important to the ethical acceptance and development of LDLT. Thus, with the aim of clarifying this issue and providing doctors and patients a reference to consider the risk-benefit balance, we conducted this study.

Currently, no satisfactory treatment is available to eradicate HCV infection; HCV recurrence is an important outcome related to the long-term survival of patients and usually occurrs around 0.5-1.5 years after LT [24, 50]. Whether the difference of donor types influenced the recurrence rate after transplantation was controversial. Related studies [51–54] indicated a rapid regeneration of hepatocytes caused by a compensatory regenerative process after LDLT, and better human leukocyte antigen (HLA) matching between donor and recipient might facilitate intrahepatocyte HCV proliferation; the former was verified in vitro, where a higher HCV internal ribosome entry site activity and replication were found in actively dividing cells [55, 56]. Contrarily, some other studies [57–59] thought less immunosuppression dose and acute cellular rejection (ACR) after LDLT might reduce HCV recurrence rate. In our study, the synthesis of the aforementioned factors showed a similar recurrence rate between LDLT and DDLT.

Whether HCC recurrence is more frequent in LDLT remains controversial. Independent risk factors of HCC recurrence after LT for HCC included tumor size (exceeding 5 cm in diameter), low-grade histologic differentiation, and gross invasion of major hepatic vessels [25]. Furthermore, Park et al. [33] believed that LDLT itself was an independent risk factor of HCC recurrence. At present, the following reasons were thought to cause a higher HCC recurrence in LDLT. First, with a short and inadequate waiting time before LDLT, the aggressiveness of tumor biology might not be readily recognized and clinically undetectable micrometastases or vascular invasion might not become apparent; this is called the “test of time” [37, 60–62]. As a result, a higher recurrence rate was induced in LDLT recipients than in DDLT recipients who had a relatively longer waiting time. This hypothesis was supported by some studies. Kulik and Abecassis [63] found a 15% HCC recurrence rate in patients with a T_1_ or T_2_ stage in the short waiting time group that was sharply in contrast to 0% in patients with T_3_ or T_4_ tumors in the long-waiting-time group. Moreover, significant activation of cell signaling pathways which led to tumor migration and invasion in small size grafts was demonstrated to promote tumor growth and metastasis after LT in an animal study [64]. Second, the regeneration of liver grafts is a natural course after LDLT [65]. However, the rapid regeneration of the liver might release more upregulation factors such as growth factors and cytokines, which might establish a favorable environment for tumor progression in cases of persistent occult extrahepatic tumor foci and accelerate the growth of tumor cells; this progress finally increases the HCC recurrence rate in LDLT [66, 67]. Third, it was thought that LDLT was more likely to result in acute phase graft injury in low graft recipient weight ratio LT, which might lead to cell adhesion, angiogenesis, and migration and provide a more favorable environment for the growth of tumor cells [68, 69]. Fourth, the greater bile duct and hepatic artery length and the preservation of vena cava in LDLT recipients might leave residual tumor or violate the tumor capsule; the greater manipulation of the livers might also lead to tumor embolus detachment through the hepatic veins [44]. Although numerous theories support the fact that LDLT recipients suffered a higher HCC recurrence rate, slight but not significantly lower 1-, 3-, and 5-HCC recurrence rates in LDLT were observed in our study. It was confusing, and some opposite factors must work in this process. Further studies at the molecular or genetic level are needed.

Postoperative biliary complications have been commonly viewed as the “Achilles heel” of LT [70], and concern about an increased risk for biliary complications in LDLT has been a worry. Although a short CIT and decreased ACR in LDLT could reduce the occurrence of biliary complications [58, 71, 72], numerous studies still indicate higher biliary complications in LDLT recipients for technical factors. Some studies have shown that biliary complications could be decreased dramatically with increased experience [40, 73–75]. We have seen this also in the subgroup analysis; the OR of the biliary complication rate of a bigger sample size group was much lower than that of a smaller sample size group. It indicated that greater experience was critical in considerably minimizing the technical complications in LDLT. Except for the learning curve, other possible explanations for a higher biliary complication rate in LDLT included inferior quality of the LDLT grafts [76–82], high frequency of double or multiple biliary anastomoses, high Model for End-stage Liver Disease (MELD) score associated with relatively inadequate arterial perfusion [83], and dislodgement of the biliary drainage tube or biliary leakage after removal of the tube [84]. Fortunately, biliary complications might not be lethal in most situations for the application of radiological interventions [85].

Postoperative vascular complications, especially hepatic artery problems, were another major surgical morbidity. In our study, LDLT was associated with a significantly higher rate of vascular complications. This might be due to insufficient length for reconstruction, smaller vessel diameter, and greater risk of a twist of the vascular pedicle of LDLT grafts [86]. Different from biliary complications, vascular complications did not decrease as centers gained greater experience. This might indicate that the higher vascular complication rate was mainly caused by the difference of donor types itself and could not be reduced with an accumulation of experience. Further studies are needed to explain this issue.

In our study, pooled patient OS were significantly better in LDLT recipients. It might be related to a better quality of living grafts and better conditions of the patients when receiving LT. Most studies showed that the mean donor age was significantly higher in DDLT, while the use of grafts from donors older than 40 to 50 years of age has been proven to result in poor patient survival [87, 88]. Simultaneously, DDLT groups had notably higher MELD scores and longer waiting time; this generally resulted in a more debilitated overall state by the time the patients received LT [10] and finally had a negative impact on patients' survival. Moreover, ACR, which was less in LDLT for better HLA matching, was also identified as a negative factor in DDLT [35, 89–91]. It is worth highlighting here that OS of LDLT patients improved dramatically as centers' LDLT experience increased. When detecting significant heterogeneity in the synthesis of OS by LDLT size (<100 or ≥100), the ORs of OS were significantly greater in subgroups with a bigger size. It indicated that centers' LDLT experience greatly influenced the patient's OS and “learning curve” might contribute in OS. Improvements in patient selection and technical advances might account for the improved OS in experienced centers.

Besides, LDLT had a significantly shorter CIT and lower postoperative intra-abdominal bleeding rate. A long CIT was related to late biliary complications [71, 72] and was a significant predictor of the overall risk of graft failure [40], while a short CIT could reduce the severity of hepatocellular injury in the early postoperative period as measured by the peak serum aspartate aminotransferase and alanine aminotransferase levels [31]. Lower postoperative intra-abdominal bleeding rate in LDLT might be due to the higher quality of living graft. Furthermore, whether a difference of coagulation function existed in different donor types was worth investigating deeply. Notably, perioperative mortality did not decrease in LDLT though there was a significantly lower postoperative intra-abdominal bleeding rate.

However, we have to acknowledge some limitations in our study. First, the definition of some complications were not clear or uniform in different studies. Second, the existence of significant heterogeneity in several outcomes could not be explained well enough by subgroup analysis. Third, included studies were conducted in different regions where policies and ethics about LT were different, and this might cause potential bias. Moreover, studies based on databases were included in our meta-analysis, and this might cause unknown overlapping of data.

In conclusion, this meta-analysis represents the latest and the most comprehensive comparison of LDLT and DDLT. Our study demonstrated that LDLT was not inferior to DDLT in consideration of RBC transfusion, length of hospital stay, perioperative mortality, retransplantation rate, HCV recurrence rate, and HCC recurrence rate, but it was an improvement in CIT, postoperative intra-abdominal bleeding rate, and OS. Therefore, LDLT remains a valuable option for patients in need of LT for it provides an excellent alternative to DDLT; the application of LDLT should be considered more especially in areas with an extremely limited deceased donor pool. However, there is a significantly higher incidence of biliary and vascular complications associated with LDLT. Further refinement in biliary and vascular reconstruction techniques and the accumulation of LT centers' experience are the key factors in expanding the application of LDLT.

## Figures and Tables

**Table 1:** Characteristics of included studies.

Reference	Year	Country	Study design	Sample size		Recipient age		Recipient diagnosis	Donor age		Follow-up period	Gender: male/female	
				LDLT	DDLT	LDLT	DDLT		LDLT	DDLT		LDLT	DDLT
Humar et al. [10]	2019	American	Retrospective cohort	245	592	NA	NA	Mixed	NA	NA	2009-2019	145/100	414/178
Fisher et al. [11]	2009	American	Prospective cohort	107	465	48.5 ± 12.0	51.5 ± 8.6	Mixed	NA	NA	1998-2009	66/41	366/99
Jiang et al. [12]	2013	China	Retrospective cohort	70	191	NA	NA	HBV related	NA	NA	2002-2009	62/8	162/29
Kim et al. [13]	2017	Korea	Retrospective cohort	109	76	52.0 ± 8.5	53.1 ± 11.0	Mixed	NA	NA	2010-2014	81/28	50/26
Hu et al. [14]	2015	China	Retrospective cohort	389	6471	48.05 ± 8.65	50.09 ± 9.43	HCC related	NA	NA	1999-2009	360/29	5819/652
Jain et al. [15]	2011	American	Retrospective cohort	35	65	50.5 ± 7.4	49.9 ± 6.8	HCV related	34.3 ± 9.3	47.2 ± 19.8	2000-2003	23/12	52/13
Ninomiya et al. [16]	2015	Japan	Retrospective cohort	133	362	57.6 ± 7.1	58.3 ± 7.4	HCC related	33.7 ± 9.6	51.7 ± 18.3	2002-2010	77/56	285/77
Sandal et al. [17]	2015	American	Retrospective cohort	62	108	52.93 ± 9.39	52.03 ± 10.58	Mixed	NA	NA	2000-2001	36/26	76/32
Hoehn et al. [18]	2014	American	Retrospective cohort	715	14282	NA	NA	Mixed	NA	NA	2007-2012	421/294	9752/4530
Macías Gómez et al. [19]	2009	Argentina	Retrospective cohort	30	357	NA	NA	Mixed	NA	NA	1995-2006	NA	NA
Thuluvath and Yoo [20]	2004	American	Retrospective cohort	764	1470	49.7 ± 5.2	49.8 ± 10.8	Mixed	35.8 ± 10.4	38.9 ± 18.1	1988-2001	434/330	836/634
Terrault et al. [21]	2014	American	Prospective cohort	195	180	NA	NA	HCV related	NA	NA	1998-2009	134/61	130/50
Schmeding et al. [22]	2007	Germany	Retrospective cohort	20	269	55.7 ± 8.9	51.4 ± 9.8	HCV related	44.2 ± 12	38.6 ± 15.2	1997-2005	12/8	164/105
Garcia-Retortillo et al. [23]	2004	Spain	Prospective cohort	22	95	NA	NA	HCV related	NA	NA	2000-2003	13/9	58/37
Bozorgzadeh et al. [24]	2004	American	Retrospective cohort	35	65	50.7 ± 7.2	50.0 ± 7.0	HCV related	34.6 ± 9.7	49.2 ± 20.4	2000-2003	23/12	52/13
Hwang et al. [25]	2005	Korea	Retrospective cohort	237	75	50 ± 8	49 ± 7	HCC related	NA	NA	1992-2002	196/41	60/15
Sotiropoulos et al. [26]	2007	Germany	Retrospective cohort	45	55	55.0 ± 10.1	53.4 ± 9.1	HCC related	NA	NA	1998-2006	33/12	42/13
Al-Sebayel et al. [27]	2015	Saudi Arabia	Retrospective cohort	222	269	NA	NA	Mixed	NA	NA	2001-2013	139/83	153/116
Xiao et al. [28]	2014	China	Retrospective cohort	84	276	NA	NA	HCC related	NA	NA	1999-2012	78/6	247/29
Vakili et al. [29]	2009	American	Retrospective cohort	28	74	NA	NA	HCC related	NA	NA	1999-2007	21/7	NA
Sandro et al. [30]	2009	Italy	Retrospective cohort	25	154	NA	NA	HCC related	NA	NA	2000-2007	NA	NA
Reichman et al. [31]	2013	Canada	Retrospective cohort	145	145	54.2 ± 7.5	53.9 ± 7.7	Mixed	NA	NA	2001-2009	117/28	117/28
Azoulay et al. [32]	2016	France	Retrospective cohort	75	576	54.2 ± 7.6	56.3 ± 7.4	HCC related	NA	NA	2000-2009	62/13	499/77
Park et al. [33]	2014	Korea	Retrospective cohort	166	50	52.5 ± 7.7	54.3 ± 9.6	HCC related	NA	NA	1999-2010	131/35	29/21
Wan et al. [34]	2014	China	Retrospective cohort	40	80	48.6 ± 9.7	49.5 ± 8.9	HCC related	NA	NA	2007-2010	34/6	68/12
Gallegos-Orozco et al. [35]	2009	American	Retrospective cohort	32	168	54 ± 9	53 ± 6	HCV related	35 ± 12	40 ± 16	1999-2008	22/10	128/40
Liu et al. [36]	2006	China	Prospective cohort	124	56	NA	NA	Mixed	NA	NA	2000-2004	97/27	44/12
Kulik et al. [37]	2012	American	Prospective cohort	100	97	55.2 ± 8.0	53.9 ± 8.5	HCC related	NA	NA	1998-2003	75/25	76/21
Li et al. [38]	2011	China	Retrospective cohort	128	221	42.96 ± 8.57	44.55 ± 9.71	Mixed	33.53 ± 9.08	32.81 ± 7.34	2005-2011	108/20	179/42
Russo et al. [39]	2004	American	Retrospective cohort	279	3955	NA	NA	HCV related	NA	NA	1999-2002	165/114	2808/1147
Freise et al. [40]	2008	American	Prospective cohort	384	216	49.6 ± 10.7	51.4 ± 9.7	Mixed	NA	NA	1998-2003	222/162	128/88
Shah et al. [41]	2007	Canada	Retrospective cohort	154	350	NA	NA	Mixed	NA	NA	2000-2006	95/59	NA
Lai et al. [42]	2009	American	Retrospective cohort	86	403	50.6 ± 12.2	53.6 ± 10.8	Mixed	NA	NA	2000-2006	42/44	289/114
Selzner et al. [43]	2008	Canada	Retrospective cohort	46	155	NA	NA	HCV related	NA	NA	2000-2005	33/13	119/36
Chen et al. [44]	2014	China	Retrospective cohort	47	94	NA	NA	HCC related	NA	NA	2007-2012	44/3	88/6
Al-Sebayel et al. [45]	2007	Saudi Arabia	Retrospective cohort	45	77	NA	NA	Mixed	NA	NA	2001-2007	29/16	38/39
Bhangui et al. [4]	2011	France	Prospective cohort	36	120	54 ± 7	56 ± 8	HCC related	NA	NA	2000-2009	32/4	100/20
Sher et al. [46]	2011	American	Retrospective cohort	34	251	NA	NA	HCV related	NA	NA	NA	NA	NA
Shen et al. [47]	2011	China	Retrospective cohort	38	67	NA	NA	HCC related	NA	NA	2007-2008	33/5	62/5

**Table 2:** Newcastle-Ottawa quality assessment scale for cohort studies.

Reference	Selection	Comparability	Outcome	Total
Humar et al. [10]	4	2	3	9
Fisher et al. [11]	4	1	3	8
Jiang et al. [12]	3	2	3	8
Kim et al. [13]	4	1	2	7
Hu et al. [14]	4	1	2	7
Jain et al. [15]	3	2	3	8
Ninomiya et al. [16]	4	1	3	8
Sandal et al. [17]	4	1	2	7
Hoehn et al. [18]	4	2	3	9
Macías Gómez et al. [19]	3	0	3	6
Thuluvath and Yoo [20]	4	1	3	8
Terrault et al. [21]	4	2	3	9
Schmeding et al. [22]	3	2	3	8
Garcia-Retortillo et al. [23]	4	2	3	9
Bozorgzadeh et al. [24]	3	1	3	7
Hwang et al. [25]	4	2	3	9
Sotiropoulos et al. [26]	4	2	3	9
Al-Sebayel et al. [27]	3	1	3	7
Xiao et al. [28]	4	1	2	7
Vakili et al. [29]	4	0	3	7
Sandro et al. [30]	4	2	3	9
Reichman et al. [31]	3	2	3	8
Azoulay et al. [32]	4	2	3	9
Park et al. [33]	4	2	3	9
Wan et al. [34]	4	2	3	9
Gallegos-Orozco et al. [35]	3	2	3	8
Liu et al. [36]	4	2	3	9
Kulik et al. [37]	4	1	3	8
Li et al. [38]	3	2	3	8
Russo et al. [39]	4	2	2	8
Freise et al. [40]	3	2	3	8
Shah et al. [41]	3	2	3	8
Lai et al. [42]	3	1	3	7
Selzner et al. [43]	3	2	3	8
Chen et al. [44]	4	1	2	7
Al-Sebayel et al. [45]	3	2	3	8
Bhangui et al. [4]	4	2	3	9
Sher et al. [46]	3	1	3	7
Shen et al. [47]	4	1	2	7

**Table 3:** Subgroup analysis.

Outcomes	Subgroup	Studies (*n*)	Effect estimate (95% CI)	*P* value	Heterogeneity	Inconsistency with the overall results
CIT	DDLT < 400	3	-371.10 (-373.60, -368.60)	*P* < 0.00001	*P* = 0.20, *I*^2^ = 37%	
	DDLT ≥ 400	3	-372.02 (-445.71, -298.34)	*P* < 0.00001	*P* < 0.00001, *I*^2^ = 97%	
	Overall	6	-373.39 (-399.41, -347.37)	*P* < 0.00001	*P* < 0.00001, *I*^2^ = 96%	
Vascular complication rates	LDLT < 100	3	1.05 (0.51, 2.16)	*P* = 0.89	*P* = 0.24, *I*^2^ = 30%	Equivalent
	LDLT ≥ 100	3	2.89 (1.62, 5.16)	*P* = 0.0003	*P* = 0.26, *I*^2^ = 26%	
	Overall	6	2.00 (1.31, 3.07)	*P* = 0.001	*P* = 0.11, *I*^2^ = 45%	
Biliary complication rates	LDLT < 100	6	3.71 (1.58-8.71)	*P* = 0.003	*P* < 0.0001, *I*^2^ = 82%	
	LDLT ≥ 100	8	1.77 (1.27, 2.48)	*P* = 0.0008	*P* = 0.0008, *I*^2^ = 72%	
	DDLT < 100	5	4.80 (2.88-7.98)	*P* < 0.00001	*P* = 0.10, *I*^2^ = 49%	
	DDLT ≥ 100	9	1.74 (1.24-2.43)	*P* = 0.001	*P* < 0.0001, *I*^2^ = 77%	
	HCC related	2	2.21 (1.72, 2.83)	*P* < 0.00001	*P* = 0.16, *I*^2^ = 50%	
	Not HCC related	12	2.21 (1.46, 3.34)	*P* = 0.0002	*P* < 0.00001, *I*^2^ = 79%	
	Overall	14	2.23 (1.59, 3.13)	*P* < 0.00001	*P* < 0.0001, *I*^2^ = 77%	
HCV recurrence	DDLT < 100	2	1.92 (0.95, 3.87)	*P* = 0.07	*P* = 0.20, *I*^2^ = 39%	
	DDLT ≥ 100	2	0.62 (0.12, 3.15)	*P* = 0.56	*P* = 0.006, *I*^2^ = 87%	
	Overall	4	1.10 (0.39, 3.10)	*P* = 0.86	*P* = 0.001, *I*^2^ = 81%	
1-year HCC recurrence	LDLT < 100	3	0.56 (0.35, 0.91)	*P* = 0.02	*P* = 0.21, *I*^2^ = 35%	Favors LDLT
	LDLT ≥ 100	5	1.29 (0.62, 2.71)	*P* = 0.49	*P* = 0.0002, *I*^2^ = 81%	
	Overall	8	1.00 (0.61, 1.66)	*P* = 0.99	*P* = 0.0007, *I*^2^ = 72%	
3-year HCC recurrence	LDLT < 100	2	0.73 (0.46, 1.14)	*P* = 0.17	*P* = 63, *I*^2^ = 0%	
	LDLT ≥ 100	3	0.99 (0.44, 2.23)	*P* = 0.98	*P* = 0.0002, *I*^2^ = 89%	
	Overall	5	0.86 (0.52, 1.41)	*P* = 0.54	*P* = 0.001, *I*^2^ = 78%	
5-year HCC recurrence	LDLT < 100	4	0.70 (0.50, 0.98)	*P* = 0.04	*P* = 0.56, *I*^2^ = 0%	Favors LDLT
	LDLT ≥ 100	4	1.19 (0.49, 2.90)	*P* = 0.70	*P* < 0.00001, *I*^2^ = 91%	
	Overall	8	0.87 (0.54, 1.38)	*P* = 0.55	*P* < 0.00001, *I*^2^ = 81%	
1-year OS	LDLT < 100	9	1.27 (0.90, 1.77)	*P* = 0.17	*P* = 0.15, *I*^2^ = 33%	Equivalent
	LDLT ≥ 100	9	1.43 (1.02, 2.01)	*P* = 0.04	*P* = 0.0002, *I*^2^ = 74%	
	HCC related	9	1.68 (1.19, 2.37)	*P* = 0.003	*P* = 0.05, *I*^2^ = 49%	
	Not HCC related	9	1.07 (0.87, 1.32)	*P* = 0.50	*P* = 0.14, *I*^2^ = 35%	Equivalent
	Overall	18	1.32 (1.01, 1.72)	*P* = 0.04	*P* = 0.0004, *I*^2^ = 61%	
3-year OS	LDLT < 100	8	1.17 (0.87, 1.58)	*P* = 0.29	*P* = 0.70, *I*^2^ = 0%	Equivalent
	LDLT ≥ 100	7	1.52 (1.18, 1.95)	*P* = 0.001	*P* = 0.02, *I*^2^ = 60%	
	HCC related	9	1.55 (1.17, 2.04)	*P* = 0.002	*P* = 0.08, *I*^2^ = 43%	
	Not HCC related	6	1.21 (0.98, 1.50)	*P* = 0.07	*P* = 0.75, *I*^2^ = 0%	Equivalent
	Overall	15	1.39 (1.14, 1.69)	*P* = 0.0010	*P* = 0.05, *I*^2^ = 41%	
5-year OS	LDLT < 100	10	1.20 (0.95, 1.52)	*P* = 0.13	*P* = 0.22, *I*^2^ = 24%	Equivalent
	LDLT ≥ 100	6	1.49 (1.02, 2.16)	*P* = 0.04	*P* < 0.0001, *I*^2^ = 81%	
	HCC related	10	1.43 (1.01, 2.03)	*P* = 0.05	*P* = 0.0003, *I*^2^ = 71%	
	Not HCC related	6	1.15 (0.92, 1.42)	*P* = 0.21	*P* = 0.64, *I*^2^ = 0%	Equivalent
	Overall	16	1.33 (1.04, 1.70)	*P* = 0.02	*P* < 0.0001, *I*^2^ = 66%	

## Abbreviations

ACR: Acute cellular rejection
CI: Confidence interval
CIT: Cold ischemia time
DDLT: Deceased donor liver transplantation
DRO: Duration of the recipient operation
HCC: Hepatocellular carcinoma
HCV: Hepatitis C virus
HLA: Human leukocyte antigen
LDLT: Living donor liver transplantation
LT: Liver transplantation
MD: Mean difference
MELD: Model for end-stage liver disease
OR: Odds ratio
OS: Overall survival
RBC: Red blood cell.

## Supplementary Materials

Supplementary Materials We submitted supporting information to help improve and prove the reporting quality of our study. These include the PRISMA 2009 checklist and the PRISMA 2009 flow diagram.
