# Supplementary material for: Increased Surgical Complications but Improved Overall Survival with Adult Living Donor Compared to Deceased Donor Liver Transplantation: A Systematic Review and Meta-Analysis
Source: Biomed Res Int. 2020 Aug 24;2020:1320830. doi: 10.1155/2020/1320830 (PMC7468609; doi:10.1155/2020/1320830)
Supplement: Supplementary Materials — We submitted supporting information to help improve and prove the reporting quality of our study. These include the PRISMA 2009 checklist and the PRISMA 2009 flow diagram. [file 1320830.f1.zip › PRISMA-2009-flow-diagram.doc]

**PRISMA 2009 Flow Diagram**

**Screening**

**Included**

**Eligibility**

**Identification**

Records identified through database searching
(n = 1485 )

Additional records identified through other sources
(n = 0 )

Records after duplicates removed
(n = 1439 )

Records screened
(n = 1439 )

Records excluded
(n = 1390 )

Full-text articles assessed for eligibility
(n = 49 )

Full-text articles excluded, with reasons
(n = 10 ):

Overlapping cohorts (n=2);

Single-arm studies (n=5);

Without available data (n=1);

Pediatric LT (n=1);

Combined liver and kidney transplantation (n=1)

Studies included in qualitative synthesis
(n = 39 )

Studies included in quantitative synthesis (meta-analysis)
(n = 39 )
